# Identification of immunogenic KIF5B-RET fusion neopeptides driving immune stimulation in tumor specific CD8+ T cells

**DOI:** 10.3389/fimmu.2025.1635810

**Published:** 2025-10-30

**Authors:** Micah B. Castillo, Sakuni Rankothgedera, Shiyanth Thevasagayampillai, Aaranyah Kandasamy, Jaila Lewis, Cole Woody, Martiela Vaz de Freitas, Dinler Amaral Antunes, Randa El-Zein, Preethi H. Gunaratne

**Affiliations:** ^1^ Department of Biology and Biochemistry, University of Houston, Houston, TX, United States; ^2^ UH Sequencing Core, Department of Biology & Biochemistry, University of Houston, Houston, TX, United States; ^3^ Department of Medicine, Houston Methodist Research Institute, Houston, TX, United States; ^4^ Human Genome Sequencing Center, Baylor College of Medicine, Houston, TX, United States

**Keywords:** RNA fusions, chimeric RNAs, neoantigens, immunopeptides, KIF5B-RET fusion, precision immunotherapy, cancer vaccine

## Abstract

**Introduction:**

Non-classical neoantigens at the fusion junctions of chimeric RNAs are tumor- specific with a low risk of autoimmunity and therefore represent ideal targets for personalized vaccines. We present a platform to discover immunogenic neoantigens that drive CD8+ T cell clonotypes from chimeric RNA fusion junctions to promote tumor-reactive T cell expansion and prevent tumor recurrence following immunotherapies.

**Methods:**

RNA sequencing data from 15 Lung Adenocarcinoma and 15 Squamous Cell Carcinoma patients (tumor and adjacent normal tissues) were analyzed. The KIF5B [Exon 1-15] | RET [Exon 12- 19] fusion was selected from a patient-derived xenograft (PDX) model based on its established role as an actionable cancer driver in an independent tumor with the same junction. We assessed the affinity of neopeptides from the KIF5B-RET fusion to MHC Class I molecules using in silico tools MHCNuggets and MixMHCPred 2.

**Results:**

HLA-C07:02 showed the highest affinity for 9-mer peptideswith NNDVKEDPK, which emerged as the strongest binder based on HLA-Arena docking and binding energy calculations. Immunogenicity was evaluated by IFNg Enzyme-Linked Immunosorbent Spot (ELISpot) assays using HLA-C07:02- matched Peripheral Blood Mononuclear Cells (PBMCs) from two donors. CD8+ T cells from both donors responded to specific junction peptides. Single-cell 5’gene expression RNA sequencing and T Cell receptor mapping of activated T cells identified 15 TCR clonotypes, five of which had high activation. Key residues in CDR3a and CDR3b are crucial for CD8+ T cell activation. NNDVKEDPK and KEDPKWEFP showed minimal cross-reactivity with the normal tissues.

**Discussion:**

This study demonstrates a robust pipeline for identifying and validating immunogenic neoantigens from chimeric RNAs to design personalized cancer vaccines with high immunogenicity and low cross-reactivity.

## 1 Introduction

Tumor-associated neoantigens accumulated during cancer progression have been a growing focus of vaccine development in the past decade. Studies assessing neoantigen load have observed strong correlations with clinical responses to immunotherapy (1) as well as high somatic mutational burden. Many candidate neoantigens have also been shown to improve survival in patients treated with immune checkpoint blockades for non-small cell lung cancer (NSCLC) (2) and melanoma (3, 4). Here, we present a platform that can extract non-classical neoantigens from fusion junctions of chimeric RNAs generated through structural variants from chromosomal translocations, inversions, and deletions, as well as transplicing events following read-through transcription of neighboring genes. The novel junctions in the fusion proteins generated from chimeric RNA products provide reservoirs of tumor-specific neopeptides that are selected for immunogenicity using HLA-matched Peripheral Blood Mononuclear Cells (PBMCs) and low autoimmunity based on a comprehensive screen of the normal immunopeptidome of humans.

In a study by The Cancer Genome Atlas (TCGA) completed in 2020, a set of recurrent canonical fusions was identified and defined as “actionable” based on the availability of a drug approved by the FDA or in various stages of clinical trials available to target one or both gene partners. Fourteen genes, including RET, were found to be part of the canonical fusions targeted by 36 drugs in 21 different cancers (5). We screened for actionable fusions in non–small cell lung cancer (NSCLC), which accounts for nearly 80% of lung cancer cases and exhibits a median survival of less than one year following diagnosis (6). NSCLC can be divided into three main subtypes: adenocarcinoma (LUAD), squamous cell carcinoma (LUSC), and large-cell carcinoma. In this study, we focused on the KIF5B-RET fusion protein, which is sensitive to vandetanib, a multi-kinase inhibitor (7). Several fusions have been reported in LUAD, including that of the kinesin family member 5B-RET proto-oncogene (KIF5B-RET) (8). Identified as a chromosomal inversion in the liver metastases of an NSCLC patient in 2011, it has since been found in 1-2% of lung adenocarcinoma patient cohorts (7). KIF5B-RET gene fusion results from chromosomal inversion between the long and short arms of chromosome. Four central fusion junction variants have been reported: KIF5B [exon15] – RET [exon 12], KIF5B [exon 16] – RET [exon 12], KIF5B [exon23] – RET [exon12], and KIF5B [exon14] – RET [exon 12]. These KIF5B-RET variants are not expressed in normal lung tissue but are highly expressed in certain adenocarcinoma lung cancer tissues. This fusion protein has been reported to be responsible for overactive tyrosine kinase activity in lung adenocarcinomas expressing protein. Due to this expression, changes are observed in the morphology of the cells along with increased proliferation, similar to the Kirsten Rat Sarcoma viral oncogene homolog (KRAS) V12 mutant present in other cancer types (8).

Previous studies have shown that cancer cells can efficiently present their own antigens and act as antigen-presenting cells (9). This study aimed to use peptides to prime the immune system to recognize and respond to MHC Class 1 presented neoantigenic peptide sequences from the KIF5B-RET fusion protein expressed in LUAD cancer patients. To do this, we leveraged several in silico prediction pipelines to identify which neopeptides generated by the junction of the KIF5B-RET protein are bioinformatically predicted to be the best binders to available MHC Class-1 alleles, with the lowest potential cross-reactivity with peptides expressed in normal tissues across the body. *In-vitro* validation of selected peptides and peptide pools was performed using an Enzyme-Linked Immunosorbent Spot (ELISpot) assay with MHC Class-1 matched PBMCs. The top expanded TCR clonotypes in fusion junction peptide-stimulated CD8+ T cell populations were identified using scRNA-seq and TCR sequencing.

## 2 Materials and methods

### 2.1 Sample cohorts

#### 2.1.1 Patient samples

Fifteen lung adenocarcinoma and fifteen lung squamous cell carcinoma specimens, each matched to an adjacent normal frozen tissue sample, were procured from the Houston Methodist Biorepository under an IRB-approved protocol. The cohort comprised 12 female and 18 male patients, ranging in age from 54 to 80 years. Tumor and adjacent normal regions were delineated on hematoxylin & eosin–stained sections by a board-certified pathologist, who selected only areas entirely devoid of histologic evidence of malignancy. The RNA‐Seq data underlying this study is available in the NCBI Gene Expression Omnibus at http://www.ncbi.nlm.nih.gov/geo/ and can be accessed with accession number GSE159857 (10).

#### 2.1.2 Patient-derived xenograft samples

Overgrown tumor tissue was sourced from the patient-derived xenograft (PDX) company XenoSTART.

### 2.2 RNA isolation and next generation sequencing

RNA from patient samples was isolated from fresh-frozen, pathologist-marked regions via laser-capture microdissection to eliminate any potential admixture with tumor cells was extracted using the Qiagen miRNeasy micro kit, followed by library preparation using the QIAseq Stranded Total RNA library preparation kit (Qiagen). RNA was extracted from the PDX tissue block using the Qiagen miRNeasy mini kit, and sequencing libraries were prepared using the QIAseq Stranded mRNA Library Preparation Kit (Qiagen). All libraries were assessed for quality using a High-Sensitivity D5000 chip on an Agilent 4200 TapeStation and quantified with a Qubit 4 fluorometer (Thermo Fisher Scientific). Libraries generated from the RNA of patients with lung cancer were sequenced on the NextSeq 500 at 20 million paired-end reads per sample, whereas the libraries generated from the PDX tissue paired-end were sequenced at >50 million reads per sample on a NovaSeq 6000.

### 2.3 Sequence alignment and fusion detection

All fusions identified within the adjacent normal samples were removed from the background. Fusions appearing only in tumor samples called by all fusion callers together were considered positive hits. Only split reads were considered when identifying the fusion genes within a particular sample. To improve the clinical relevance of the identified fusion genes, an additional filter was implemented by identifying specific actionable fusion gene partners identified in The Cancer Genome Atlas (TCGA) dataset (5). RNA-seq fastq data were aligned using several fusion calling pipelines as detailed below. The pipelines were chosen based on their specificity (confidence) in their calls or sensitivity (fusion detection rate).

#### 2.3.1 CLC genomics workbench 20 (Qiagen)

Illumina sequencing adaptors were trimmed, and reads were mapped to the human reference genome hg38 Refseq (RRID: SCR_0034^9^6) GRCh38.p9 from the Biomedical Genomics Analysis Plugin 20.0.1 (Qiagen). RNA fusions were detected using the detection fusion gene algorithm, which identifies fusion events based on the number of fusion junction-crossing reads and fusion-spanning reads. The refined fusion gene tool was used to re-count the number of fusion junction crossing reads, and the novel RNA-seq reads were mapped against a fusion reference created in the initial detection fusion gene pipeline. Only fusion split (soft-clipped) reads were considered when identifying fusion genes, as fusion spanning (discordant) reads increased the probability of false-positive fusion calls.

#### 2.3.2 Illumina Dragen RNA

Illumina sequencing adaptors were trimmed, and reads were mapped to the human reference genome hg38, no alts, and decoys. Both “RNA Quantification” and “Gene Fusion Detection” were enabled. Alignment output files were output in BAM format. All other settings were set at default values.

#### 2.3.3 Arriba, EasyFuse

Illumina sequencing adaptors were trimmed, and reads were mapped to the human reference genome hg38. All pipelines were run according to the default protocols specified in their respective GitHub pages (11, 12).

#### 2.3.4 HLA typing of RNA sequencing data

HLA typing of patient samples (GSE159857) was carried out using the OptiType pipeline with the default settings (13). As the sample data was from precious, clinical samples, the quality of several samples was not high enough to generate a confident HLA type.

### 2.4 RT-PCR and sanger sequencing

Reverse transcription of RNA samples was used to generate cDNA from PDX tissues after RNA extraction. The cDNA was then subjected to PCR amplification across the KIF5B-RET fusion junction using forward (5’-GATGATGGCATCTTTACTAAAAG-3) and reverse (5’-CGCCTTCTCCTAGAGTTTTTC-3’) primers. DreamTaq DNA Polymerase (Cat. # EP0701) was used in the 30-cycle PCR. Amplicon size was analyzed using a High-Sensitivity DNA 1000 tape on a Tapestation 4200 (Agilent, RRID: SCR_019398). Sanger sequencing was performed at the LoneStar Laboratories, Houston, TX.

### 2.5 *In-silico* neopeptide affinity predictions

Class I MHC binding affinities for 9-mer peptides from the KIF5B-RET fusion junction region were predicted using MHCnuggets and MixMHCpred 2.2. MHCnuggets were executed as previously described (14), while MixMHCpred 2.2 was run with default settings (15). Wild-type peptides positioned two amino acids away from the fusion junction served as controls. Peptides spanning the major Open Reading Frame (ORF) generated from the KIF5B-RET fusion were analyzed. MHCnuggets predicted MHC class I binding affinities as IC50 values (nM), considering peptides with IC50 < 500 nM as strong binders and ranked them accordingly. MixMHCpred 2.2 evaluated affinity in %Rank, with a cut-off of 10% indicating strong binding. The output data from both pipelines were reviewed, and the optimal HLA Class I allele was selected for further analysis.

### 2.6 Structure-based affinity predictions and peptide docking

Eight junction-spanning peptides and two wild-type peptides from the KIF5B-RET fusion gene were subjected to structural modeling using HLA-Arena (16) and APE-GEN (17). APE-GEN generated multiple models for each peptide, predicting their binding energies to the HLA-C07:02 receptor. The receptor structure was sourced from the Protein Data Bank (PDB ID: 5VGE) and processed with the R package Bio3d (RRID: SCR_024266) to remove excess molecules and verify integrity. The rigid receptor structure was prepared via PDB2PQR (18), and energy minimization was done for the structures with Gromacs (19–21). Molecular docking was performed for all 10 peptides against the rigid and energy-minimized (EM) structures of HLA-C07:02. Workflow 0 in HLA-Arena was adapted to model each peptide-receptor complex, and APE-GEN ensemble sampling was used to calculate the binding energies for ranking the peptides.

### 2.7 Peptide – HLA modeling and electrostatic potential calculations

ChimeraX was used to visualize the peptides docked with HLA-C*07:02. The electrostatic potential (ESP) of the peptide structure was calculated, and the molecular surfaces were colored red for negative potential and white to blue for positive potential (22, 23).

### 2.8 *In-silico* off-target toxicity assessment

For each 9-mer input from the KIF5B-RET fusion gene, CrossDome (24) generated a list of unrelated self-derived peptides that may have biochemically similar profiles to 9-mer inputs, which could lead to toxicity and adverse effects in cancer immunotherapies. Additionally, CrossDome was used to yield mRNA and tissue expression patterns for each 9-mer off-target peptide associated with the 9-mer input peptides from the KIF5B-RET fusion gene.

### 2.9 Peptide library generation

The fusion peptide library comprised eight neoantigens and two wild-type 9-mer peptides from the KIF5B [exon 15]–RET [exon 12] fusion gene open reading frame. Peptides were synthesized via standard solid-phase peptide chemistry and purified using reverse-phase high-performance liquid chromatography (Thermo Fisher Scientific PEPotec). The solution was reconstituted at 1 mg/mL under sterile conditions. A standardized 9-mer peptide supplied by the manufacturer served as the negative control peptide (NCP), as this peptide had no biological significance. A Cytomegalovirus (CMV) peptide pool (Cat. # 3619-1) with 42 peptides (28 MHC class I-and 14 MHC class II-restricted) was used as a positive control.

### 2.10 Human primary cells

HLA-C*07:02 allele-matched human PBMCs from two healthy donors were acquired (STEMCELL Technologies) and stored in liquid nitrogen until use. Donors were matched to the HLA-C*07:02 allele and also expressed the following HLA alleles: Donor 1 - A*02:01, A*24:02, B*15:13, B*38:02, C*08:01. Donor 2 - A*02:01, A*11:01, B*07:02, B*67:01.

### 2.11 Culture medium

The complete media consisted of RPMI-1640 growth medium supplemented with L-glutamine (Cat. # 61870036) supplemented with 10% heat-inactivated fetal bovine serum (Cat. # F0601-050), 0.1 mmol/L nonessential amino acids (Corning; Cat. # 25-025-CI), 10ug/ml Cellmaxin (Cat. # C3319-006), and 0.5 mg/mL Amphotericin B (Cat. # 15290026).

### 2.12 *In-vitro* stimulation of PBMCs using peptides

PBMCs were retrieved from liquid nitrogen, thawed in a water bath at 37°C, and washed with culture medium warmed to 37°C as described in the primary cell thawing protocol by Stem Cell Technologies. The cells were incubated at 37°C and 5% CO_2_ for 24h (Cell Resting). After resting, the cells were seeded at a concentration of 1 × 10^6^/mL in 6-well plates with culture medium containing IL-2 (10 IU/ml), IL-7 (10 ng/ml), and IL-15 (10 ng/ml). The cells of the Non-Stimulated Control (NC) wells were not treated with any peptides but were maintained under the same growth conditions as the cells of wells treated with neoantigenic peptides. The cells in the CMV-positive control wells were treated with 1 μg/ml of the CMV peptide pool and supplemented with media and growth conditions identical to the test peptide wells. The eight neoantigenic and two wild-type 9-mer test peptides were added to their respective wells at 2 μg/ml, and the plates were incubated at 37°C and 5% CO_2_ for 4 days. On day 5, 50% of the medium was replaced with fresh medium, and cells were cultured for an additional 5 days. A second round of peptide restimulation was performed with the corresponding peptides coupled with the cytokine medium before the cells were used for the ELISpot assay.

### 2.13 Isolation of CD8+ T cells from PBMCs

On Day 13, untouched CD8+ T cells were isolated from PBMCs by magnetic negative selection using a MojoSort™ Human CD8+ T Cell Isolation Kit (BioLegend; Cat. # 480012) according to the manufacturer’s instructions.

### 2.14 IFN-γ ELISpot assay

To evaluate the peptide-stimulated CD8+ T cell immune response, IFN-γ production by cells stimulated with the predicted neoantigenic peptides was quantified using a commercially available Human IFN-γ ELISpot kit (CTL ImmunoSpot, Cellular Technology Ltd.), following the manufacturer’s instructions. The plate was read using an ELISpot reader (CTL Counter, Cellular Technology Ltd.). The cell culture medium used to incubate the cells in the ELISpot plate was augmented with the corresponding peptides and IL-2 (10 IU/ml), IL-7 (10 ng/ml), and IL-15 (10 ng/ml), which were considered significant if >20 spots/1,000,000 cells were counted, and the mean spot count was at least three-fold higher than the mean spot count of the non-stimulated control.

### 2.15 5’ v2 HT single cell RNA-seq library preparation and sequencing

CD8+ T cells were isolated from junction peptide pool stimulated and non-stimulated PBMCs, spun at 500 rpm for 5 min, washed once in PBS (without calcium and magnesium) with 0.04% BSA, and then resuspended. Cell suspensions were loaded onto a 10X Genomics chip N, following the *Chromium Next GEM Single Cell 5’ HT Reagent Kits v2 (Dual Index)* protocol (CG000423|Rev C). Modular kits for 10X Chromium Connect were used to automate library preparation from cDNA. Gene Expression (GEX) sequencing libraries were generated using the *Chromium Next GEM Automated Single Cell 5’ Reagent Kits v2* user guide (CG000384|Rev D). Libraries were assessed for quality using a High-Sensitivity D5000 chip on an Agilent 4200 TapeStation and quantified with a Qubit Flex Fluorometer (Thermo Fisher Scientific). Libraries were pooled by donor and sequenced on NovaSeq X Plus (Illumina) to obtain 150 base paired end reads.

### 2.16 Post-sequencing processing

The 10X Cloud Analysis portal was used to run CellRanger v7.1.0 on all FASTQ files. The sequencing data were aligned to the GRCh38 human reference genome.

### 2.17 Analysis of single cell gene expression data

Single-cell RNA sequencing GEX data were obtained from donors 1 and 2 under stimulated and non-stimulated conditions. Filtered matrix files from CellRanger v7.1.0 were used as input into R and converted into Seurat objects using the CreateSeuratObject function from the Seurat package (25, 26). Seurat objects were created and processed independently for each condition and donor. Each Seurat object underwent SCT normalization using the SCTransform function. Concurrently, the percentage of mitochondrial genes was regressed from each object, with a final 5% mitochondrial cutoff implemented, as observed in other studies on populations of cells from PBMCs (26). The cells were filtered based on the number of expressed features, retaining those with a feature count between 500 and 10,000. Principal Component Analysis (PCA) and UMAP dimensionality reduction were performed on each object using RunPCA and RunUMAP functions, respectively. FindNeighbors and FindClusters functions were used to define clusters in the data. The DoubletFinder (RRID: SCR_018771) package was deployed on each Seurat object to identify and remove doublets, followed by subsetting to retain only the singlet cells (27). The objects were then annotated with the relevant donor and condition metadata. Following individual processing, the objects were integrated into a single Seurat object using the Seurat package’s data-integration features.

### 2.18 Annotation of cell types

Cell types were assigned based on the expression of canonical marker genes as defined in previous studies. All T-Cells were defined based on their expression of CD3D, CD3G, and CD3E (28). CD8+ T cells were defined based on CD8 expression (28). Naïve CD8 + T-Cells were further clustered based on their expression of CCR7 and SELL (28). Exhausted CD8 + T-Cells were annotated based on their expression of PDCD1 and LAG3 (28). Cycling CD8 T Cells were identified by the expression of TOP2A and MKI67 (29). Activated CD8 T Cells were called using canonical markers of activation: IFNG, TNF, GZMB, CCL3, and CCL4 (28). CD8+/CD4+ T cells were characterized by co-expression of both CD8 and CD4 genes (30). Two Natural Killer (NK) cell populations were identified and defined by the expression of KLRC1 (29, 31). Dendritic Cells were defined using the marker gene LYZ (29). B Cells were defined by the expression of MS4A1 (29).

### 2.19 Analysis of single cell immune profiling data using Scanpy and Scirpy

Single-cell TCR sequence data were processed and analyzed using Python packages Scanpy (32) and Scirpy (33). Individual components of the integrated Seurat object, including Gene Names, Metadata, PCA, and Matrix information, were prepared to construct an. h5ad file that can be used in Scanpy. Scirpy was then used to analyze the corresponding GEX and TCR data from each sample by creating a merged object, followed by subsequent analysis as described in the “Analysis of 3k T cells from cancer” tutorial (33).

### 2.20 CDR3 sequence clustering, characterization, and alignment

The top 15 variable sequences of CDR3-α and CDR3-β chains were considered as the response-positive dataset and clustered using the methodology previously described (28). Briefly, clustering was performed in GibbsCluster 2.0, with MHC class I configurations and a specified core size of the smallest variable sequence in the positive dataset (34). CDR3 chains found only within naïve populations were used as the negative datasets. The position-specific scoring matrices (PSSMs) yielded from GibbsCluster 2.0 clustering were used to conduct a position-wise Pearson correlation between positive and negative datasets. Correlation significance was assessed using Pearson’s correlation test. Similarity and identity of variable sequences were computed by pairwise sequence alignment using Clustal Omega (RRID: SCR_001591) with standard configurations (35).

## 3 Results

### 3.1 Fusion identification

**Figure 1:**
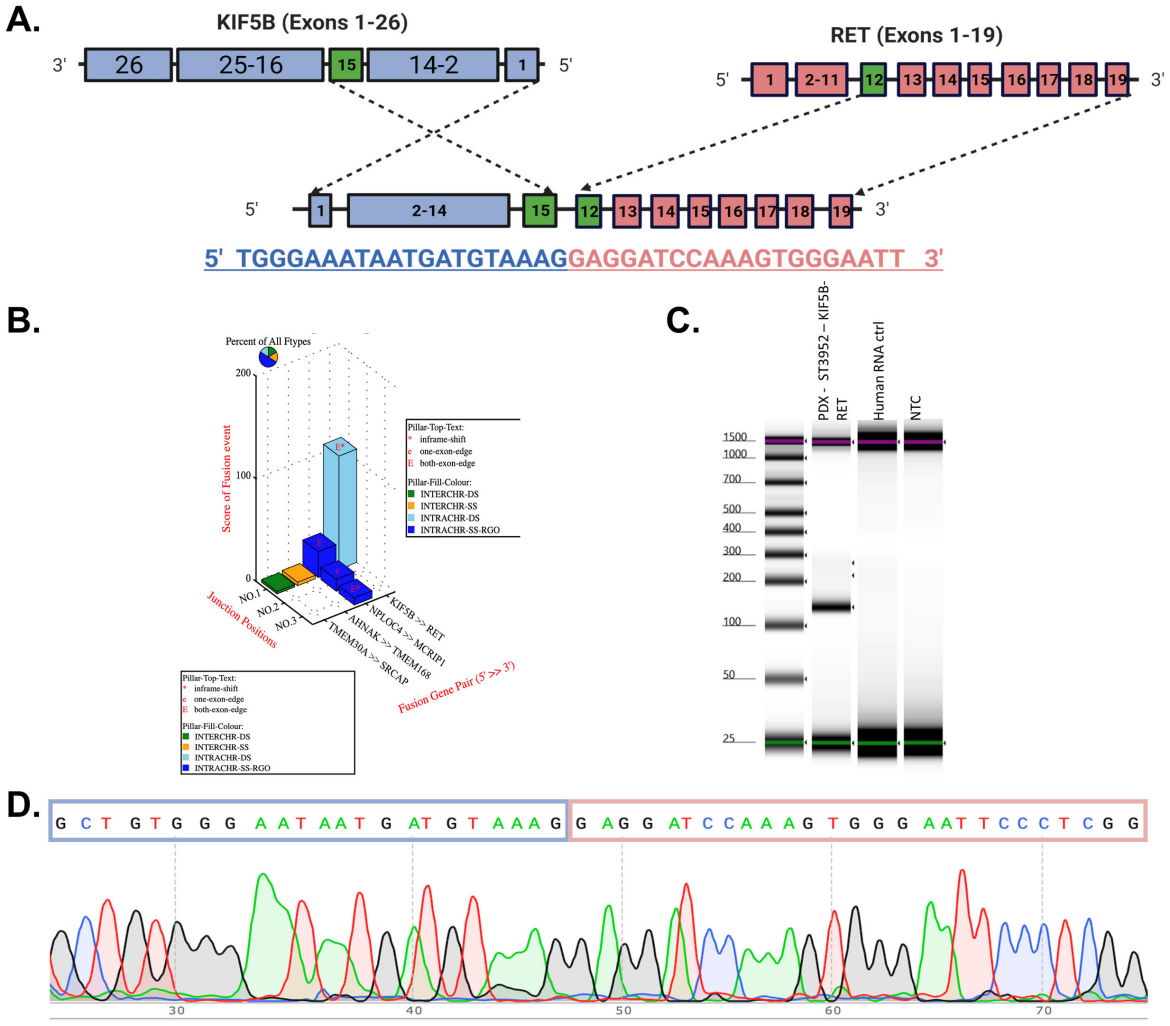
KIF5B-RET fusion identification in an LUAD Patient and validation in PDX Model by RT-PCR and Sanger sequencing. **(A)** *In-silico* identification of fusion KIF5B [Exon 15] – RET [Exon 12] from an LUAD patient sample. **(B)** In-silico fusion predictions in an LUAD PDX sample. **(C, D)** *In-vitro* confirmation of the KIF5B [Exon 15] – RET [Exon 12] junction shown by RT-PCR and Sanger sequencing.

RNA-seq data from 15 Lung Adenocarcinoma (LUAD), 15 Squamous Cell Carcinoma (LUSC) tumors, and adjacent normal samples from Houston Methodist were screened using multiple fusion callers. Each of the four fusion calling pipelines were run independently, and selection of fusions from each fusion caller were done in parallel without using any one fusion calling pipeline as the golden standard. We selected for this study, KIF5B-RET, found in a patient with LUAD and corroborated by all four fusion calling pipelines including CLC Genomics Browser Fusion Caller, Illumina Dragen Fusion Caller, Arriba, and EasyFuse(**Supplementary Table 1**). The fusion junction between exon 15 of KIF5B and exon 12 of RET is shown in **Figure 1A**. The complete fusion nucleotide sequence was constructed using the Hg38 reference sequence (**Supplementary Figure 1**), revealing 16 Open Reading Frames (ORFs), with the longest being a 935 amino acid in-frame sequence (**Supplementary Figure 2**). This fusion, previously reported in patients with LUAD with a prevalence of 1-2% was validated in previous studies and similarly identified as a chromosomal inversion by the Arriba pipeline (7, 36).

### 3.2 Validation of fusion KIF5B-RET

Due to the limited tissue from the LUAD patient with a bioinformatically confirmed KIF5B-RET fusion, the full RNA sample was used to create a sequencing library and was not confirmed using downstream methodology.

### 3.3 KIF5B-RET fusion in LUAD PDX model

The exon 15 KIF5B and exon 12 RET junction variants were also found in the LUAD patient-derived xenograft (PDX) model ST3952 from XenoSTART. This bioinformatically predicted fusion, identified across all fusion prediction pipelines, was similarly called as chromosomal inversion by Arriba (**Supplementary Figure 3**). The tissue block from XenoSTART allowed us to validate the fusion junction in the PDX model ST3952 (**Figure 1B**). In silico analysis confirmed the fusion, and *in-vitro* validation was achieved by amplifying a 131-basepair amplicon, as shown in the TapeStation Trace (**Figure 1C**). This band was absent in the normal human RNA control. RT-PCR followed by Sanger sequencing confirmed the fusion junction, which was consistent with the results of previous studies (**Figure 1D**) (7, 36).

### 3.4 Neoantigen affinity prediction identify HLA-C*07:02 binds strongly to junction peptides *in-silico*

**Figure 2:**
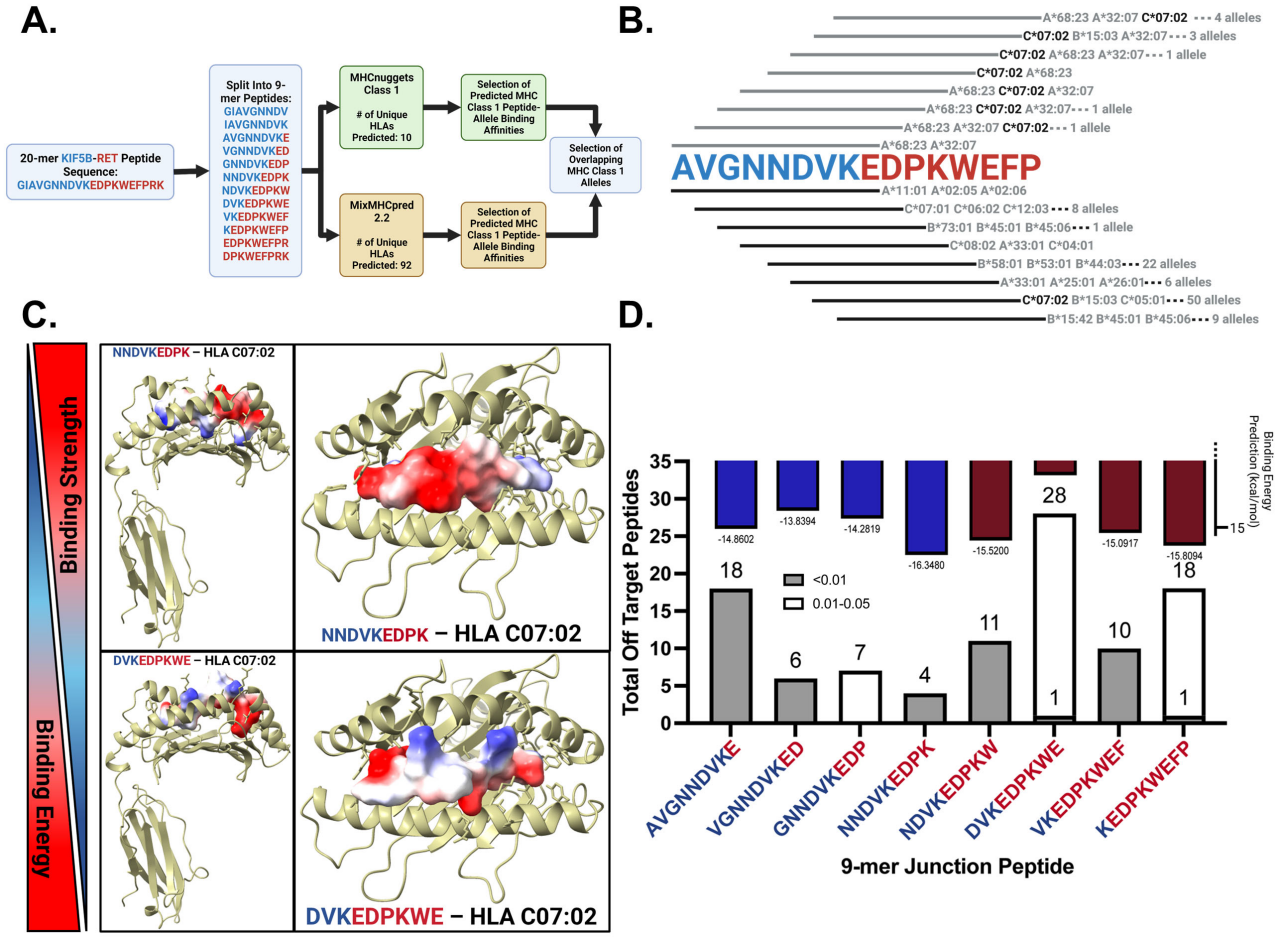
*In-silico* prediction of KIF5B – RET neopeptide binding affinities to HLA alleles and identification of possible cross-reactive responses. **(A)** Neopeptide affinity prediction pipeline. **(B)** Binding of junction peptides to MHC Class 1 alleles. Lines in grey and black correspond to the peptide sequence above and represent calls made by MHCNuggets and MixMHCPred respectively. **(C)** Peptides with highest and lowest average binding energy prediction values across three replicates. Peptides are docked in HLA-C07:02 and are colored by their electrostatic potential. **(D)** Binding energy predictions for junction peptides. Bars colored by majority contribution of amino acids in blue and red for KIF5B and RET respectively on top of the bar plot. Total number of off target peptide hits for each 9-mer junction crossing peptide shown across the bottom of the plot.

We employed MHCnuggets to predict IC50 values and MixMHCpred 2.2 to rank peptides based on motif similarity (**Figure 2A**). By comparing these two pipelines, we identified HLA allotypes with high peptide-binding affinities for potential *in vitro* validation. A %Rank cutoff of 10.00 in MixMHCpred, corresponding to IC50 values ≤ 500 nM from MHCnuggets, was used to define strong binders, as established in the literature (14, 15, 37). MHCnuggets predicted ten unique HLA class I alleles with IC50 ≤ 500 nM (**Supplementary Table 2**). Individual values for each Class I HLA allele for all junctions and wild-type peptides are shown in **Supplementary Table 3**. MixMHCpred identified 92 unique HLA class I alleles with a% rank ≤ 10 (**Supplementary Table 4**). Overlapping predictions identified a single junction peptide, “VKEDPKWEF,” which binds to four HLA Class I alleles (**Supplementary Table 5**). HLA-C*07:02 emerged as a strong binder across multiple peptides (**Figure 2B**) and was consistently predicted by both the pipelines. It showed robust binding to junction peptides but not to wild-type KIF5B peptides (**Supplementary Table 6**). Precision HLA typing of patient samples was performed using RNA-seq data from GSE159857 (**Supplementary File 2**). HLA-C*07:02 emerged as a potential HLA genotype of interest and further supported exploring our in-silico predictions. Aside from this HLA, other more prevalent HLA alleles in this cohort included HLA C*06:02 and HLA C07*01 which showed moderate binding affinity to junction peptide VGNNDVKED and were expressed by the patient carrying the KIF5B-RET fusion (**Supplementary Figure 4**).

### 3.5 Structural modeling and HLA-peptide binding strength predictions using HLA-arena

Using HLA-Arena, we investigated the binding strengths of eight junction-spanning and two wild-type peptides from the KIF5B-RET fusion gene to HLA-C*07:02, selected for its high affinity in previous predictions. HLA-Arena integrates tools for the structural modeling and analysis of peptide-HLA complexes, providing a comprehensive environment for this study.

We docked the peptides to the HLA-C07:02 rigid receptor structure using APE-GEN to generate multiple models and predict the binding energies. The crystal structure of HLA-C07:02 was prepared using the Bio3d software (RRID: SCR_024266). Ensemble sampling of each peptide-HLA complex was performed and the binding energies for the best conformations were calculated.

Electrostatic potential analysis showed that the peptide NNDVKEDPK, with the lowest binding energy, had positive electrostatic potential regions oriented towards the binding pocket, while DVKEDPKWE, with the highest binding energy, had negative electrostatic potential regions facing inward (**Figure 2C**).

**Figure 2D** (upper panel) presents the binding energy results, where lower binding energies correlate with stronger binding affinities. NNDVKEDPK, KEDPKWEFP, and NDVKEDPKW exhibit the lowest binding energies.

### 3.6 Off-target toxicity predictions of neoantigenic peptide sequences using CrossDome

One concern with peptide vaccines is the potential for cross-reactivity, in which the immune system may recognize similar peptides from host proteins, leading to autoimmune responses. To assess the cross-reactivity of neoantigenic peptides from the KIF5B-RET fusion junction, we utilized the bioinformatics tool CrossDome. CrossDome identifies cross-reactive candidates based on global sequence similarity rather than intrinsic MHC binding affinity. Therefore, to strengthen biological relevance, we integrated independent HLA-binding predictions (via HLA-Arena/NetMHCpan) with the CrossDome hits.

We analyzed eight 9-mer neoantigenic peptides crossing the fusion junction and two wild-type peptides from KIF5B and RET. Using CrossDome’s default p-value cutoff of ≤ 0.005 yielded few cross-reactivity results, with three peptides (GNNDVKEDP, DVKEDPKWE, and KEDPKWEFP) showing no cross-reactivity (**Supplementary Table 7**). We raised the p-value cutoff for lenient identification, categorizing cross-reactive hits into p-value bins of ≤ 0.01 and 0.01 - 0.05 [**Figure 2D** (lower panel)].

NNDVKEDPK had the least off-target hits, followed by KEDPKWEFP, which correlated with its high binding strength. For KEDPKWEFP, 18 of 19 off-target hits were in the 0.01 - 0.05 category, indicating low confidence in these results [**Figure 2D** (lower panel)]. By cross-referencing immunopeptidomics data with tissue expression levels, we identified cross-reactive peptides in the retina, skeletal muscle, spleen, liver, and cerebellum (**Supplementary Figure 5**).

### 3.7 Assessment of immune stimulation using the IFN-γ ELISpot assay

**Figure 3:**
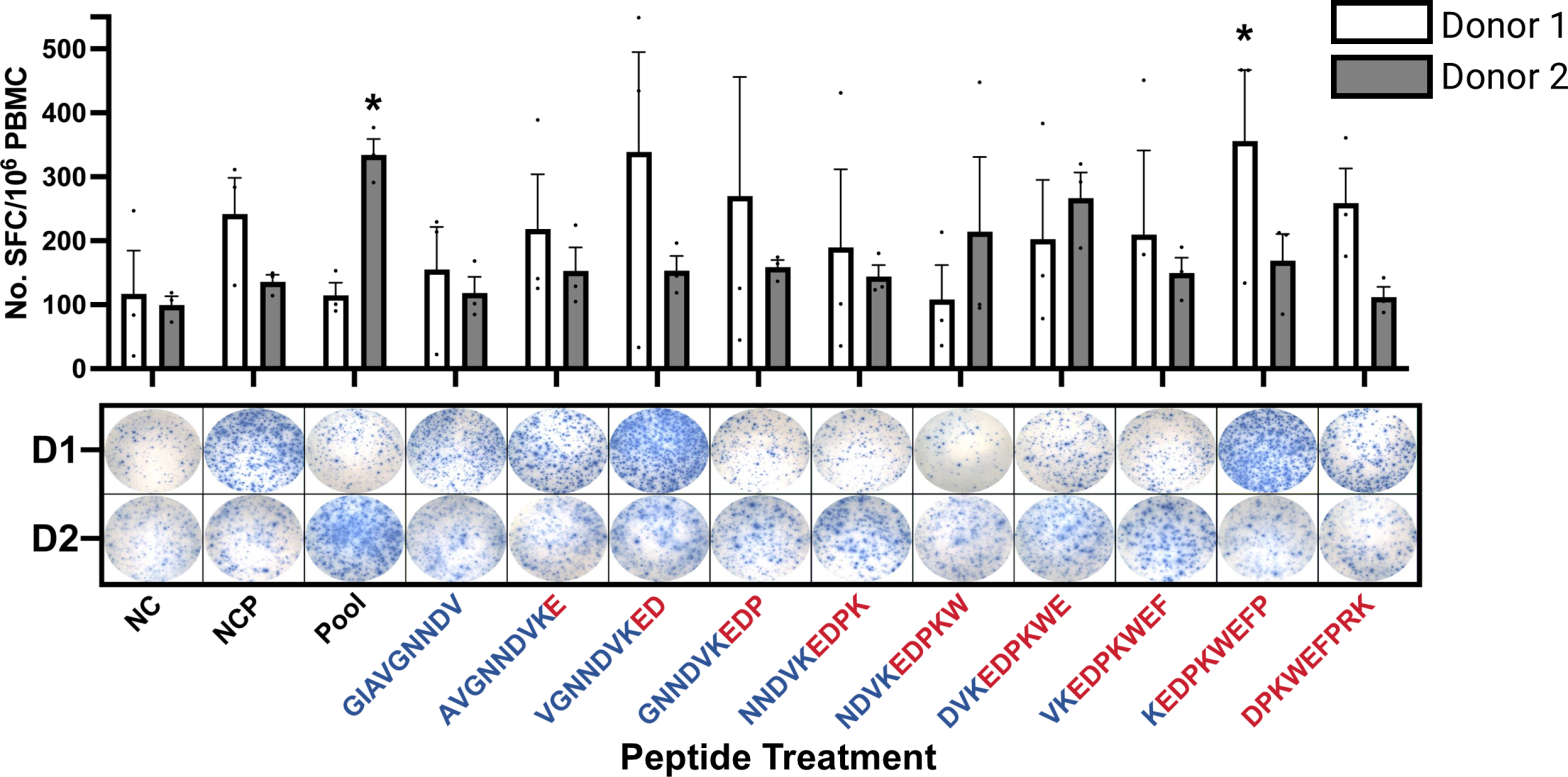
IFN-γ ELISpot of CD8+ T-Cells Stimulated with KIF5B-RET Neoantigenic Junction Peptides. NC: Non-Stimulated Control, NCP: Non-Activation Control Peptide, Pool: Neoantigen Junction Pool. T cell responses are considered positive if >20 spots/1M cells were counted, and the mean spot count was at least three-fold higher than the mean spot count of the NC. (*significantly positive T cell responses). D1/D2: Donor 1/Donor 2. Peptides colored in blue and red by representation of KIF5B or RET amino acids, respectively. Whiskers represent Standard Error of the Mean (SEM).

Isolated CD8+ T-Cells stimulated with KIF5B-RET junction peptides were assessed for activation using ELISpot assay to measure IFN-γ expression (**Supplementary Figure 6**; **Supplementary Table 8**). Variability in the immune response was observed among donors, with certain 9-mer peptides within the neoantigenic junction sequence eliciting strong responses in one donor and weak responses in another (**Figure 3**).

For Donor 1, the peptide sequence KEDPKWEFP triggered a significant immune response compared with the negative control (NC) treatment (**Figure 3**), consistent with its second-highest predicted binding strength according to HLA-Arena[(**Figure 2D** (upper panel)]. Additionally, Donor 1 exhibited higher IFN-γ secretion when stimulated with the peptide VGNNDVKED, although this was not considered a positive stimulation in all replicates (**Figure 3**). This peptide has lower binding energies in in *in-silico* predictions.

In contrast, Donor 2 showed a positive response to the pool of junction neopeptides, particularly towards DVKEDPKWE, despite its weaker binding strength as predicted by HLA-Arena, as shown in **Figure 3**.

### 3.8 Clustering and cell type annotation in Seurat

In conjunction with our ELISpot assays, isolated CD8+ T-Cells stimulated with KIF5B-RET junction peptides were assayed via single cell transcriptomics to assess transcriptional changes within our populations of interest. Seurat was used to cluster the cells according to their respective gene expression signatures in a total of 28 unique clusters (**Supplementary Figure 7A**). The expression of canonical markers identified previously in literature was used to determine the cell populations within cells captured after peptide stimulation (**Supplementary Figure 7B, 8**). T Cells were identified based on the expression of CD3D, CD3G, and CD3E (28). Naïve CD8 T-Cells (clusters: 0, 14, 15, 17, 20, 21, and 25) were characterized by the presence of the marker genes CCR7 and SELL (28). These markers are associated with cells in a quiescent state and respond to new antigens. Exhausted CD8 T-Cells (clusters 1, 2, 3, 4, 5, 6, 7, 12, 13, 16, 22, and 24) were denoted by the expression of LAG3 and TOX (28). Cycling CD8 + T-Cells (Cluster 8), typically associated with T cell activation, were marked by the genes TOP2A and MKI67 (29). Activated CD8 T-Cells (clusters 9, 10, and 11) were identified using the markers IFNG, TNF, GZMB, CCL3, and CCL4 (28). Two distinct clusters of Natural Killer (NK) cells were identified. NK-1 (Cluster: 18) cells were characterized by the expression of KLRC1 (31) and NK-2 (Cluster: 27). NK-2 cells, the secondary and smaller subset of Natural Killer cells, expressed KLRC1 and NCAM1 (29). The expression of the LYZ gene marked Dendritic Cells found in (cluster 23), which play an integral role in antigen presentation to T cells (29). B Cells (Cluster: 26) were identified by the expression of MS4A1, a marker gene associated with B cell development and differentiation (29). CD8/CD4 T Cells (cluster 19) co-expressed CD8A, CD8B, and CD4 (30). Cluster identities were assigned based on the above markers (**Supplementary Figure 9**).

### 3.9 CD8 T cell subset and re-clustering

**Figure 4:**
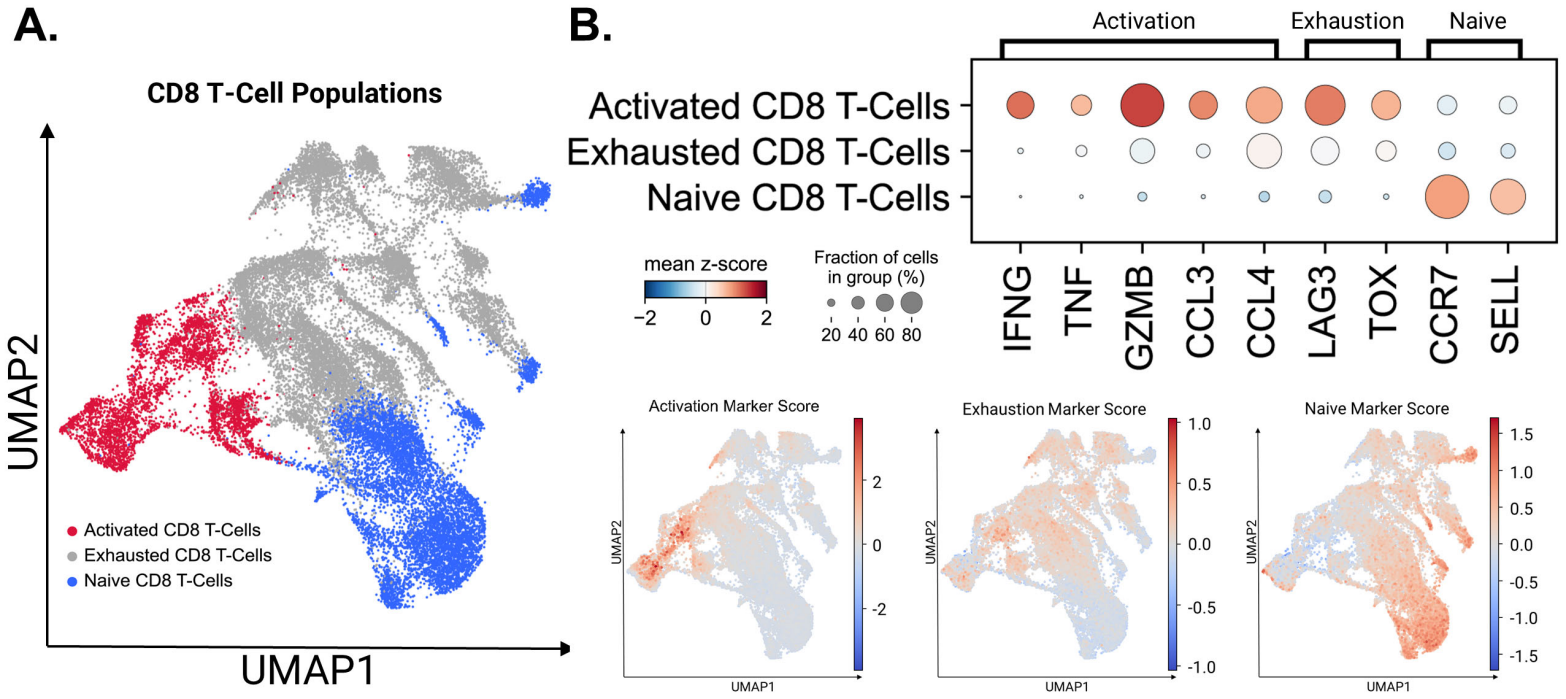
Activated, exhausted, and naive T-Cell populations identified using canonical markers. **(A)** CD8+ T cell populations identified by the expression of canonical marker genes. **(B)** The expression levels of canonical marker genes used to identify CD8+ T cell populations are represented by dot plot. Gene Score UMAPs of activation, exhaustion, and naive T Cell Markers across all CD8+ T Cell populations are shaded based on the expression scores of cell state markers.

While performing magnetic CD8 T cell isolation, it is known that there is a margin of error in the captured cells. Clusters 18, 19, 23, 26, and 27 had expression profiles that differed from those of the CD8+ T cells we originally selected for (**Supplementary Figure 7B**). These clusters correlated with the two identified NK cell populations, B cells, dendritic cells, and the population of T cells co-expressing CD4/CD8 markers. These populations were removed from the CD8+ T cell population and the final cell populations of interest were re-clustered using Seurat. Cell populations were defined using markers described previously for naïve, exhausted, and activated CD8+ T cells (**Figure 4A**).

Marker expression in the three significant CD8+ T cell populations: Activated, Exhausted and Naïve states are presented in **Figure 4B**. Although major exhaustion markers were expressed within the active population, as seen in previous studies, the diminished expression of activation markers within exhausted populations allowed us to separate them into two states (28, 29). UMAP embeddings utilizing marker scores were used to confirm our cell identity assignments across the CD8 + T cell populations. To quantify the key transcriptional programs in CD8^+^ T cells, we computed per-cell module scores with Scanpy’s sc.tl.score_genes for three gene sets. The exhaustion score was defined by LILRB1, PDCD1, LAYN, HAVCR2, LAG3, CD244, CTLA4, TIGIT, TOX, VSIR, BTLA, ENTPD1, CD160, LAIR1, and GZMK. The activation score was defined by IFNG, TNF, GZMB, and CCL3. The naïve score was defined by IL7R, CCR7, SELL, FOXO1, KLF2, KLF3, LEF1, TCF7, ACTN1, and FOXP1. Higher module-score values indicate greater enrichment of the corresponding program within individual cells (28, 38, 39).

### 3.10 Donor-specific differences observed within CD8 T cell populations

**Figure 5:**
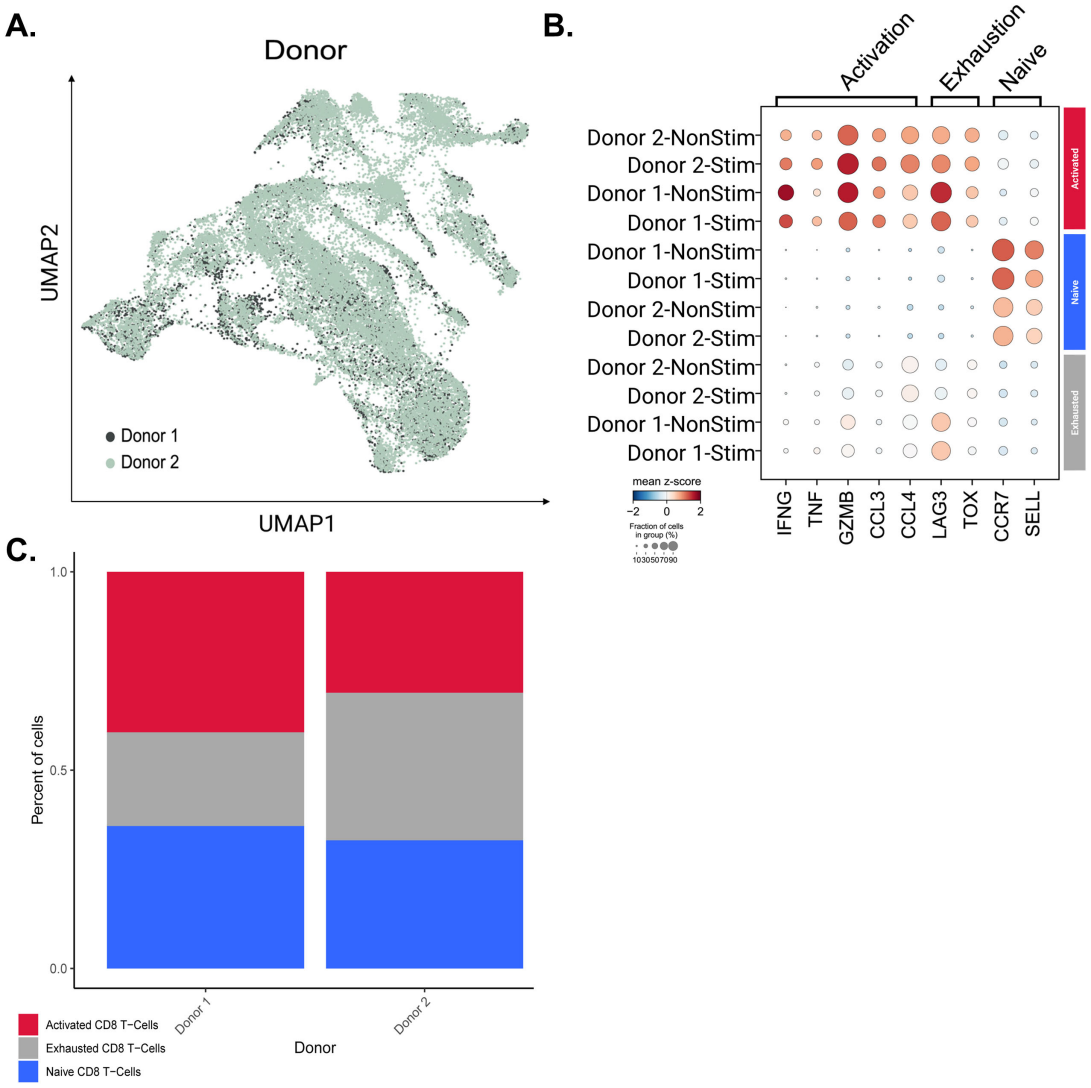
Querying donor-specific CD8 T cell population differences. **(A)** UMAP of CD8+ T cells colored by donor. **(B)** Dot plot of T cell markers across active, exhausted, and naive CD8+ T cell populations between donors. **(C)** Bar plot representing the proportional composition of each cell type across both donor’s CD8 T cell population.

As identified in our ELISpot assays, the stimulation of CD8+ T cells exhibited notable donor-specific effects in that they were stimulated at different levels by the peptides or peptide pool. This coincides with previous literature demonstrating variability in immune responses from donor to donor (40, 41). Cells from Donor 1 and Donor 2 were compared, to compare the gene expression profiles of each of the three major CD8+ T cell populations between each donor (**Figures 5A, B**).

We found a statistically significant difference in the expression of CCL4, TNF, and IFNG in active CD8+ T cells and LAG3 in exhausted CD8+ T cells (**Supplementary Figure 10**). However, while the adjusted p-values for these genes were significant and less than 0.05 (41), the LFC for each marker gene was < 1-fold in both cases. Given the slight differences in gene expression, we wanted to push the question further to identify a possible relationship with the difference in immune stimulation seen in our ELISpot assays. To this end, we divided the populations of cells by the donor and examined the percentage of each of the three major cell populations (**Figure 5C**). Both intra- and inter-donor differences were profiled in this way, making it clear that there was a decrease in the proportion of cells within the Exhausted CD8+ T cell population in Donor 1 compared to Donor 2. We also looked at the proportion of activated exhausted, and naive CD8 T cells within stimulated and non-stimulated populations for each donor (**Supplementary Figure 11**). It was observed that cell population proportions were similar within donors when comparing stimulated versus non-stimulated samples, likely resulting from cytokine stimulation by IL-2, IL-7, and IL-15 for all samples during ELISpot. These cytokines were added to propagate our PBMCs throughout the 14-day ELISpot workflow. This further prompted us to investigate any differences in T cell clonotype expansion between stimulated versus non-stimulated cells.

### 3.11 Investigation of TCR clonotype expansion across CD8 T-cell populations

**Figure 6:**
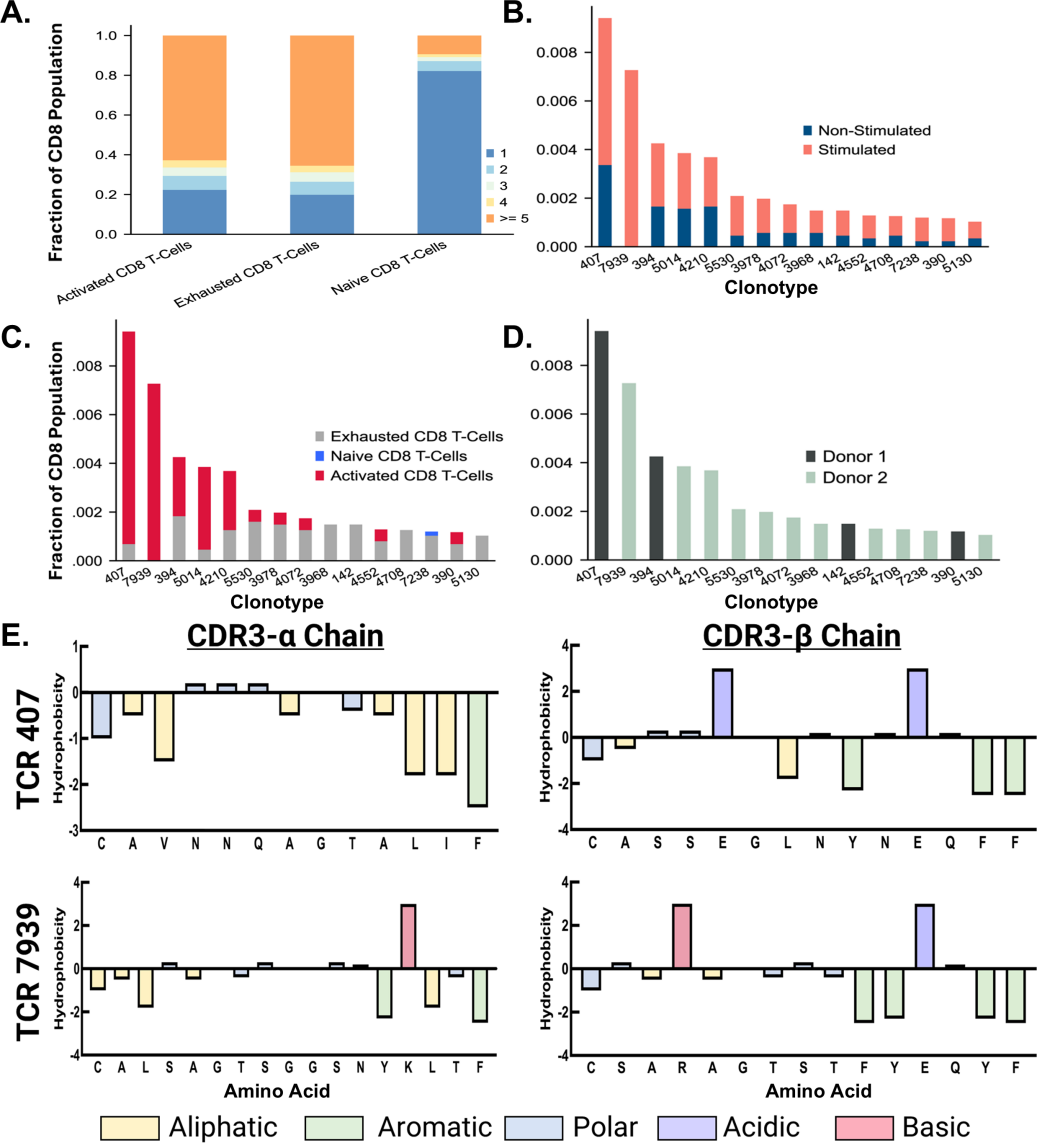
Characterization of Top Expanded TCR Clonotypes. **(A)** A bar chart shows the proportion of clonal expansion in active, exhausted, and naive populations after peptide stimulation. **(B)** The top 15 TCR clonotypes expressed primarily in stimulated CD8+ T cells are displayed in a stacked bar chart, colored by stimulated and non-stimulated status. **(C)** The top 15 TCR clonotypes expressed primarily in stimulated CD8+ T cells are displayed in a stacked bar chart, colored by cell type. **(D)** The top 15 TCR clonotypes expressed primarily in stimulated CD8+ T cells are displayed in a bar chart, colored by donor. **(E)** The CDR3-β chain of the top TCR sequences from Donor 1 and Donor 2 are denoted in dark green and light green, respectively. Residues are colored based on chemical properties.

After exploring donor-specific effects based on gene expression and changes in cell populations, we investigated clonal expansion across each of the three major cell populations identified within the CD8+ T-cell subset. The increase in clonotype count seen across naïve, exhausted, and activated populations is illustrated in (**Figure 6A**). The naïve population mainly expressed one clonotype, whereas the exhausted and active populations exhibited higher clonality. The clonotype count was calculated as the number of TCR clonotypes exhibited by more than one cell. Thus, the non-expanded naïve population is expected to have unique TCR clonotypes across cells within the population.

### 3.12 Identification and allocation of TCR clonotypes

Confirming clonal expansion across the populations of CD8+ T cells isolated after stimulation, we identified the top 15 TCR clonotypes expressed primarily by stimulated cells. This required examination of the proportions of stimulated versus non-stimulated cells expressing each clonotype. We then ranked the clonotypes based on the proportion of cells from the peptide pool stimulated samples *vs*. non-peptide-stimulated samples (**Figure 6B**). This approach allowed us to assess which TCRs explicitly responded to the junction peptide pool and not the cytokine media used during the stimulation described in the methods. Following the rank ordering of TCRs, we shifted our focus to the cell populations that predominantly expressed these TCRs. The top five clonotypes, ranked by the overall proportion of cells, were mainly found within the active CD8+ T cell population (**Figure 6C**). This highlights the possible role of these clonotypes in mediating the immune response when exposed to the KIF5B-RET junction peptide pool. In contrast, the remaining ten TCR clonotypes were predominantly correlated with exhausted cell populations, with a singular clonotype additionally being identified in the naive population. This indicates varying responsiveness and potential functional specialization among these clonotypes, potentially influencing the immune response differently.

Given the donor-specific effects observed in this study, a focused analysis was conducted to assess the prevalence of top TCRs within each donor. This aspect of the data showed that the first and second most prevalent TCR clonotypes were sourced from donors 1 and 2, respectively (**Figure 6D**). This coincides with the data from previous studies in that low rates of TCR sequence overlap were found in stimulated samples (42, 43). However, it should be noted that this assay included only two donors, and testing on more donors would be necessary to validate this finding thoroughly. Additionally, Donor 2 responded with the highest clonal expansion to the junction peptide pool, as 11 of 15 clonotypes from the top 15 TCRs were sourced from this donor. This reflects the results of our ELISpot assay, in which Donor 2 responded significantly to stimulation with a pool of junction peptides.

### 3.13 Characterization, clustering, and position based Pearson analysis of top expanded TCR sequences

**Figure 7:**
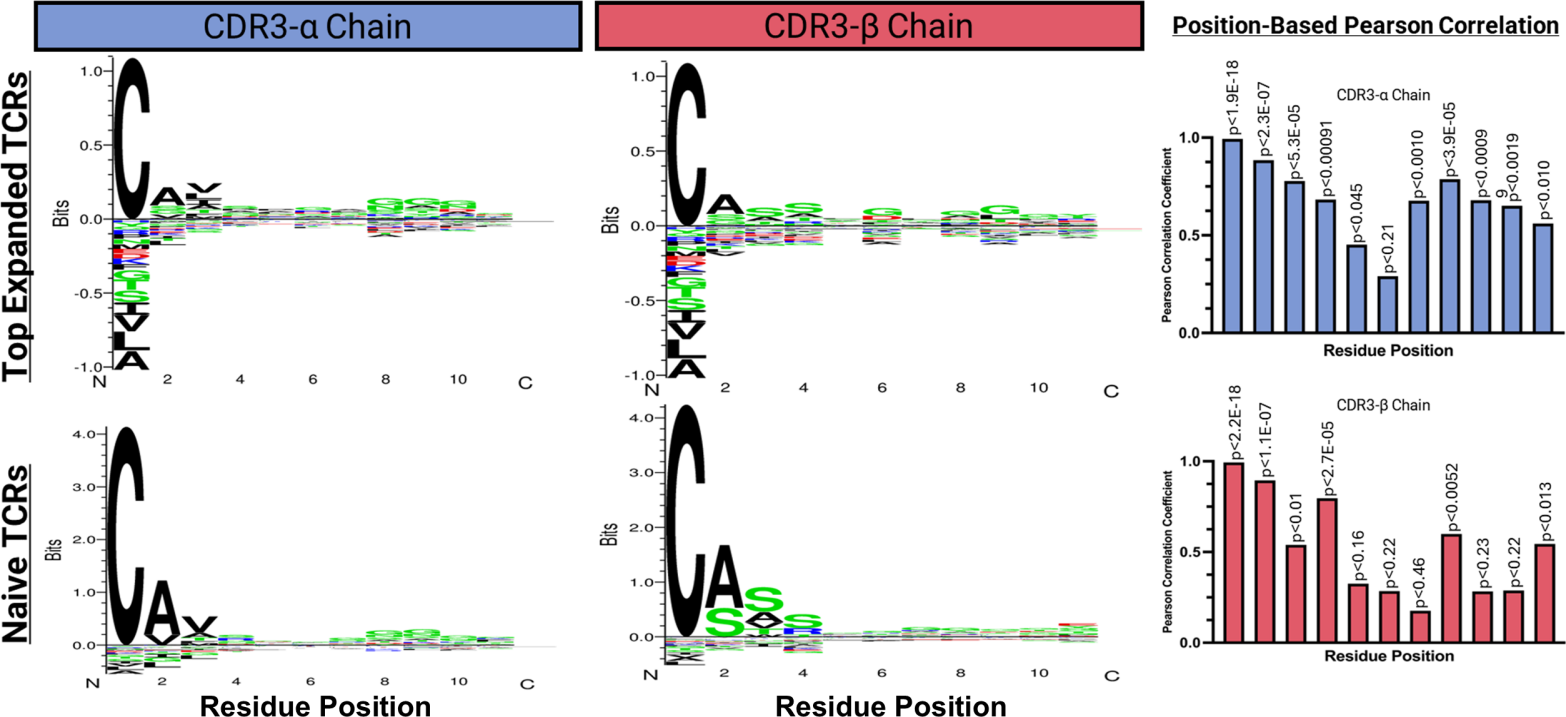
Variable sequence clustering, motif generation, and Position Based Pearson correlations (PBPC) between the top 15 expanded and naive TCR clonotypes. The online software GibbsCluster 2.0 was used to generate motifs. Underlying matrix files from the clustering were used as input to the PBPC. Amino acid positions that were found to be non-correlative between the top expanded TCR clonotypes versus naïve TCR clonotypes were identified by yielding a p ≥ 0.05.

Characterization and analysis of TCR sequence data from both donors revealed regions of conserved residues or chemical properties in the CDR3-α and CDR3-β sequences of the top expanded TCR sequences. The sequences from different donors showed noticeable chemical similarities, particularly in the regions located on the exterior of the sequence. However, they were considerably different as they progressed towards the central region. The CDR3-α chains displayed aromatic and aliphatic conservation, whereas the CDR3-β chains contained aromatic, polar, and acidic residues (**Figure 6E**). Using the online software GibbsCluster 2.0, we also performed a variable sequence clustering analysis to generate sequence motifs of the CDR3-α and CDR3-β chains from the top 15 expanded TCR clonotypes in active populations, as well as the top 15 clonotypes that appeared strictly within the naïve populations (**Figure 7**). This approach allowed us to compare the motifs between these two populations, with the final goal of identifying positions that differed. However, discerning which residues were significantly different from the motifs alone was challenging. To address this challenge, we used a Position-Based Pearson Correlation (PBPC) analysis with the underlying matrix files of the clustering as an input. Applying PBPC allowed us to identify the residues with significant differences between the CDR3-α and CDR3-β chains of the top expanded and naïve TCRs (**Figure 7**). Specifically, one position within the CDR3 alpha chain and five positions within the CDR3 beta chain were identified to have Pearson correlation coefficients with p-values greater than 0.05. This suggests a lack of correlation at these positions for each chain when comparing the top 15 expanded TCR clones and clones found within naïve populations. When comparing the conserved positions found by PBPC to a Clustal residue alignment and conservation assessment, the overlapping positions were found to be rich in glycine residues (**Supplementary Figure 12**). This glycine-rich characterization provides crucial insight into the distinctive nature of the top expanded TCR sequences compared to the naïve sequences. Additionally, when looking at the top expanded TCR sequences, we can highlight specific clonotypes that respond to the junction peptide pool of the KIF5B-RET fusion gene.

## 4 Discussion

### 4.1 The KIF5B [exon 15] – RET [exon 12] fusion gene was identified in LUAD

Gene fusions found within tumor tissues, primarily those driving tumor growth, provide an area of emerging interest for cancer treatment and prevention. Significant advancements in next-generation sequencing technologies and bioinformatics pipelines have made it easier to explore this aspect and identify possible targets. This study identified an actionable gene fusion, KIF5B-RET, created by the fusion of exons 1–15 of KIF5B and 12–19 of RET in LUAD patient samples and an independent LUAD PDX model. In addition, this fusion gene has been previously reported (7) as a chromosomal inversion on chromosome 10, which was confirmed in our LUAD PDX sample, highlighting its potential applicability in targeted therapeutic interventions.

Kohno et al. provided comprehensive insights into the origins and functionalities of the fusion protein resulting from the KIF5B-RET gene fusion event (7). The findings from this study revealed that the fusion leads to an overactive tyrosine kinase due to the loss of crucial domains of the RET protein, which morphologically resembles a KRASV12 mutant phenotype characterized by unrestrained cellular proliferation.

Further studies across a much larger cohort of lung adenocarcinoma patient tissue samples add to our knowledge of RET fusions by investigating the prevalence of RET gene fusion partners. They found that the predominant partner was KIF5B (36). This fusion has a consistent appearance rate across studies, typically 1-2% in patients with LUAD, emphasizing its significance as a recurrent actionable mutation.

One of the significant goals of classical oncogenic science is to identify common mutations that serve as broad therapeutic targets for the most significant patient cohort. However, the relatively unique and less prevalent nature of mutations, such as the oncogenic driver KIF5B-RET fusion, should not deter studies from examining it, but instead, prompt the development of personalized treatments for each cancer type.

The identification of the KIF5B-RET fusion gene within a subset of LUAD patients not only aligns with previous findings but also paves the way for the development of personalized treatment methods in the form of peptide or mRNA vaccines targeting the junctions of the fusion proteins and the neopeptides created by them.

Recent evidence has supported that gene fusions generate novel junction peptides that are absent from the normal proteome, minimizing central tolerance and making them attractive vaccine targets provided the junctional epitopes can be displayed by the patient’s HLA alleles. Fusion-derived epitopes have been shown to bind common HLA class I molecules and elicit T-cell responses as seen with EML4-ALK-derived, HLA-A*02:01-restricted cytotoxic T lymphocytes (44). Immunopeptidomics has also provided direct mass-spectrometric evidence of HLA-bound peptides arising from oncogenic fusion proteins like BCR-ABL (45). These data establish biological plausibility that fusion junctions are naturally processed and presented in human tumors.

Clinical feasibility in human patients has also been tested. In the phase-I FusionVAC22_0 trial, individuals whose tumors harbor the DNAJB1-PRKACA fusion are treated using a junction-derived peptide vaccine (Fusion-VAC-XS15) combined with anti-PD-L1 (atezolizumab), explicitly employing HLA-restricted fusion-neoantigen vaccination (46). In parallel, the first in-human personalized mRNA neoantigen vaccines utilized each patient’s peptides restricted to their own HLA repertoire. This vaccine has shown to expand antigen-specific T cells with durable CD8^+^ memory reported on extended follow-up. This study provides a strong clinical precedent that patient-specific, HLA-restricted neoantigen vaccination is feasible and immunogenic in humans (47, 48). Taken together, these mechanistic data and clinical trials support our working hypothesis that a patient’s KIF5B-RET fusion can yield HLA-presentable junctional epitopes suitable for inclusion in a cancer vaccine contingent on restricting predictions to the patient’s HLA type and validating top candidates where feasible.

### 4.2 *In-silico* predictions of junction peptide binding affinity assist in identifying HLA-C*07:02, best peptide binders, and lowest cross-reactivity to wild type peptides

To assess the affinity of the junction neopeptides formed by the KIF5B-RET fusion gene expressed and presented by the tumor, we employed two sequence-based *in-silico* HLA affinity prediction pipelines and a pipeline to examine Class I HLA affinity from a structural standpoint (9, 49). HLA-C*07:02 was identified as the best binder *in silico*—however, several other HLA alleles aligned with binding affinities that were close in score. Along with the rarer HLA-C allele class, the best ranking results in the more common HLA- A and -B classes were HLA-A*68:23 and a tie between HLA-B*45:01 and HLA-B*45:06 (50). In terms of junction neopeptides from KIF5B-RET being incorporated into a future peptide vaccine, this is a positive *in silico* finding. The ability of these neopeptides to bind to a broader range of HLA alleles will increase the applicability of this vaccine to cancer patients expressing the KIF5B-RET fusion. We moved forward with the allele HLA-C*07:02 because of its strong affinity and predicted a preference for binding to tumor-derived peptides.

In addition to assessing affinity, understanding the structural features of peptide-HLA binding is essential for determining whether the peptide will be presented. Insights from HLA-Arena revealed the significance of electrostatic complementarity in the efficient binding of peptides to HLA molecules, emphasizing the importance of the positioning and orientation of peptides in the HLA-binding cleft (51–53).

While investigating cross-reactivity, noticeable variability among neopeptides was discovered, stressing the importance of sequence as well as structure in a peptide’s capability to cross-react. Some peptides, such as NNDVKEDPK, showed strong binding and minimal off-target hits, which requires further validation through *in-vitro* assays. While the data gained from these tools may provide an excellent look into what may occur *in-vitro*, we also observed several limitations. These tools, albeit efficient, may not emulate all the biological variables present in living systems. The written algorithms and pre-existing measurements used to create these pipelines comprised only known variables. Thus, there may be components that have not been well explored in the existing literature that are unaccounted for. The fundamental understanding of the efficacy, safety, and off-target effects of neoantigenic peptides will stem from rigorous *in-vitro* experiments, which will allow for the validation of *in-silico* findings, identification of false positives or negatives, and assurance of the efficacy and safety profile of any specific neoantigen.

Combining sequence-based affinity predictions with structural considerations gave us a multimodal look at neoantigen affinity and cross-reactivity. Using these pipelines, we were able to efficiently develop a neoantigen-based vaccine against LUAD tumor cells expressing the KIF5B-RET fusion gene. Additionally, this methodology can be quickly and affordably inserted into a pipeline to validate fusion peptides for other cancer types. However, we must stress the importance of validating these data *in-vitro* to corroborate the results of the *in-silico* data. Notably, precision HLA typing of patient RNA-seq samples is a highly informative technique which would yield a more personalized approach to peptide vaccine development. Utilization of the HLA typing results from patient RNA-seq data could narrow our search for an HLA of interest in future studies.

### 4.3 *In-vitro* assessment of immune stimulation by KIF5B-RET junction peptides identifies donor-specific immune responses

This study utilized the ELISpot assay to measure the immune response after stimulation with KIF5B-RET neopeptides. Along with standard controls seen in the literature, we also used wild-type peptides from the KIF5B and RET genes as negative controls to further substantiate any positive results (28, 54, 55). This assay also demonstrated well-documented aspects of donor variability in peptide-based vaccines, with immune reactions varying distinctly between donors. Although some peptides were effective in provoking immune responses in one donor, they failed to produce similar results in another. These results push for more personalized treatment methods instead of the “one size fits all’.

The comparative analysis of *in-silico* predictions and *in-vitro* results allowed us to validate that while predictive tools, such as those used in this study, offer good preliminary insights, the biological responses may differ. This is not to say that these pipelines are entirely off the mark, as the second-best binder *in-silico* was the best CD8 + T-cell activator *in-vitro*.

The data obtained from this study provide evidence of the utility of *in-silico* predictive tools, but also highlight the role of *in-vitro* validations, developed controls, and the understanding of donor-specific variations. It also contributes to the development of a complete pipeline to probe the stimulation of the immune system using immunogenic neopeptides from the junction of fusion proteins caused by mutations within a cancer subtype.

### 4.4 Single cell RNA sequencing with immune profiling reveals TCR sequences and conserved residues which correlate with immune response against KIF5B-RET junction neopeptides

We used single-cell sequencing in partnership with immune profiling of T Cell Receptors to identify specific TCR clonotypes within CD8+ T cell populations responsive to stimulation by a pool of neopeptides from the junction of the KIF5B-RET fusion protein.

Examination of individual immunological fingerprints revealed donor-specific responses, with variations in the expression of exhaustion markers, such as LAG3, possibly affecting the overall immune response (28). It became evident that each donor’s immunological makeup influences how their CD8+ T cells react to stimuli, following previous literature, and further emphasizes the relevance of individual differences in immune system functions (40, 43). These data further reinforce the potential of the KIF5B-RET fusion junction neopeptides as targets for therapeutic approaches, such as peptide and mRNA vaccines.

When focusing specifically on the sequences of the CDR3-α and CDR3-β chains, we were able to identify conserved positions within each chain that were unique to the TCRs of the stimulated cell populations. We also found conserved glycine residues unique to these positions, as observed in previous studies of T-cell responses (56, 57). The data from this study defined TCR sequences and sequence motifs, revealing key positions and residues that elevate the probability of an immune response against specific peptides within the KIF5B-RET fusion junction. This information can be used to improve future therapeutic strategies, including adoptive T-Cell therapies utilizing TCR engineering. This paves the way for more personalized and effective treatment options for LUADs expressing the KIF5B-RET fusion.

In summary, the culmination of the data covered in this study contributes to the field of oncogenomics and immunology for the development of HLA-matched peptide vaccines to target tumors carrying oncogenic fusion KIF5B-RET. It also opens new avenues for developing patient-specific therapeutic strategies using the pipeline established to predict and prevent disease recurrence in a wide range of cancer types.

## Data Availability

The original contributions presented in the study are publicly available. This data can be found via the European Nucleotide Archive (ENA) under accession: PRJEB100809.
